# Fatal Infection with Murray Valley Encephalitis Virus Imported from Australia to Canada, 2011

**DOI:** 10.3201/eid2302.161161

**Published:** 2017-02

**Authors:** Daniel J. Niven, Kevin Afra, Mircea Iftinca, Raymond Tellier, Kevin Fonseca, Andreas Kramer, David Safronetz, Kimberly Holloway, Michael Drebot, Andrew S. Johnson

**Affiliations:** University of Calgary, Calgary, Alberta, Canada (D.J. Niven, M. Iftinca, R. Tellier, K. Fonseca, A. Kramer, A.S. Johnson); Fraser Health Authority, Surrey, British Columbia, Canada (K. Afra);; Provincial Laboratory for Public Health, Calgary (R. Tellier, K. Fonseca);; Public Health Agency of Canada, Winnipeg, Manitoba, Canada (D. Safronetz, K. Holloway, M. Drebot)

**Keywords:** Murray Valley encephalitis virus, viruses, Murray Valley encephalitis, arbovirus, encephalomyelitis, meningitis/encephalitis, flaviviridae, flavivirus, traveler, imported case, fatal infection, Northern Territory, Australia, Canada

## Abstract

Murray Valley encephalitis virus (MVEV), a flavivirus belonging to the Japanese encephalitis serogroup, can cause severe clinical manifestations in humans. We report a fatal case of MVEV infection in a young woman who returned from Australia to Canada. The differential diagnosis for travel-associated encephalitis should include MVEV, particularly during outbreak years.

In 2011, an outbreak of 17 confirmed cases of Murray Valley encephalitis (MVE) occurred in Australia, mostly in Western Australia and Northern Territory, where the virus is considered enzootic in water fowl (*1*). We report a travel-associated case of MVE detected in Canada related to that outbreak.

## The Case-Patient

A previously healthy 19-year-old woman from Alberta, Canada, returned from Australia with increasing fatigue, fever, lethargy, and confusion. She had spent 6 months in New Zealand as part of an agricultural exchange, in which her activities included hay bundling, heavy machinery operation, and manure management but no direct zoonotic contacts.

Before returning to Canada, she took a solo 10-day vacation in Australia. Her itinerary and activities were reconstructed from tickets, receipts, and email correspondence. She flew to Darwin, in the tropical region (Top End) of Northern Territory, and then left on a 3-day tour of Kakadu, Mary River, and Litchfield National Parks. She then took a bus to Alice Springs, where she spent another 3 days touring local sites, after which she flew from Alice Springs to Calgary, Alberta, Canada, through Sydney, Australia; Auckland, New Zealand; and Los Angeles, California, USA. She was not previously vaccinated for Japanese encephalitis virus.

Symptoms of excessive fatigue developed the day of her return to Canada (day 1) and were initially attributed to jet lag. The next day, she was drowsy, confused, and febrile. The patient was admitted to a rural community hospital where empiric high-dose intravenous ceftriaxone, vancomycin, and acyclovir were administered.

Because of progressive neurologic deterioration, the patient was airlifted to Foothills Medical Centre in Calgary. On arrival (day 3), she was febrile (temperature 39.7°C) and lethargic and showed worsening confusion, incomprehensible speech, inappropriate verbal responses, and a fluctuating level of consciousness. At examination, she had mild tachypnea. She did not have nuchal rigidity, focal neurologic signs, or a rash. Initial clinical tests results are shown in Table 1. Test results for malaria were negative. Chest radiography showed fine, diffuse, interstitial markings.

**Table 1:** Clinical data for a patient with a fatal infection of MVEV imported from Australia to Canada, 2011*

Variable	Reference range or value	Day 3	Day 5†
Blood			
Leukocyte count, × 10^9^ cells/L	4.0–11.0	11	
Hemoglobin, g/L	120–160	101	
Platelet count, × 10^9^/L	150–400	111	
Sodium, mmol/L	133–145	139	
Potassium, mmol/L	3.3–5.1	3.8	
Chloride, mmol/L	98–111	108	
Carbon dioxide, mmol/L	21–31	22	
Random glucose, mmol/L	3.3–11.0	6.2	
Creatinine, μmol/L	35–100	61	
Total bilirubin, μmol/L	0–24	8	
Alanine aminotransferase, U/L	1–40	15	
Lipase, U/L	0–80	298	
Cerebrospinal fluid			
Leukocyte count, × 10^6^ cells/L	0.5–5.0	45	142
Neutrophils, %	0	3	36
Lymphocytes, %	70	74	60
Monocytes, %	30	3	4
Xanthochromia	NA	None	None
Glucose, mmol/L	2.2–3.9	4.0	3.5
Total protein, g/L	0.15–0.45	0.33	0.92
Gram stain	NA	Negative	Negative

*MVEV, Murray Valley encephalitis virus; NA, not applicable. †All blood test results for day 5 were within reference ranges.

**Figure 1:**
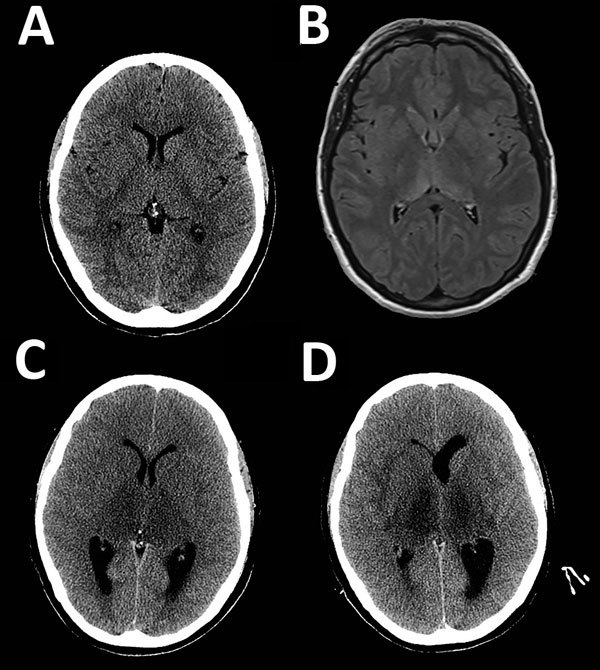
Neuroimaging during course of illness for a patient with a fatal infection of Murray Valley encephalitis virus imported from Australia to Canada, 2011. Each image corresponds to an axial cross-section through the thalamus and basal ganglia. A) Computed tomography (CT) at day 3. B) Magnetic resonance imaging (T2 flipped attenuation inversion recovery sequence) at day 3 showing abnormalities in the posterior thalami and splenium of the corpus callosum. C) CT when a fixed, dilated, right pupil (day 8) developed in the patient showing marked thalamic hypo-density and obstructive hydrocephalus. D) CT before death (day 10) showing necrosis of both thalami and a dilated left lateral ventricle.

Results of computed tomography (CT) of the brain were within reference ranges (Figure 1, panel A). Results of cerebrospinal fluid (CSF) testing were abnormal (Table 1). Gadolinium-enhanced magnetic resonance imaging showed areas of restricted diffusion in the splenium of the corpus callosum and T2 flipped attenuation inversion recovery sequence hyperintensity in the posterior aspects of both thalami (Figure 1, panel B). A provisional diagnosis of flavivirus encephalitis was made. High-dose intravenous meropenem was given because of possible melioidosis encephalomyelitis, which has been reported in the Top End of Northern Territory (*2*).

The patient became increasingly agitated and had worsening hypoxemia, which required transfer to the intensive care unit for tracheal intubation and mechanical ventilation. Results of repeat chest radiography were consistent with development of the acute respiratory distress syndrome. On day 4, she began to show decerebrate posturing with increased deep tendon reflexes, diffuse rigidity, unresponsiveness, and a downward gaze preference. Repeat CT of the brain showed evolving hypodensity of both thalami with extension into the brainstem.

On day 5, a presumptive diagnosis of MVE was made on the basis of results obtained for CSF by reverse transcription PCR. Primers specific for flavivirus nonstructural protein 5 coding region (*3*) yielded a 770-nt sequence obtained from a 863-bp amplicon, which showed 98% identity with that of Murray Valley encephalitis virus (MVEV) strain 1–51 (GenBank accession no. AF161266).

Serum samples collected on day 4 and tested by using an ELISA were positive for MVEV IgM but negative for IgG and neutralizing antibodies (Table 2). CSF abnormalities were most pronounced on day 5 (Table 1), after which time CSF cell counts decreased rapidly. All cultures of blood, urine, CSF, and respiratory secretions were negative for MVEV. A broad investigation into possible etiologies was conducted for blood, saliva, CSF, brain tissue, and respiratory samples (Table 2).

**Table 2:** Laboratory test results for a patient with a fatal infection of MVEV imported from Australia to Canada, 2011*

Sample (collection day) and test	Result
Cerebrospinal fluid (3)	
Enterovirus RT-PCR	Negative
West Nile virus RT-PCR	Negative
Herpes simplex virus type 1 and 2 PCR	Negative
Varicella zoster virus PCR	Negative
MVEV RT-PCR†	Positive
Intrathecal antibody production	ND
Saliva (4)	
Rabies virus RT-PCR	Negative
Blood (4)	
HIV 1 and HIV-2 antibody	Negative
Antibodies against arbovirus antigens‡	Negative
MVEV§	Positive
Rickettsial antibody	Negative
Syphilis antibody	Negative
Cryptococcal antigen	Negative
Hepatitis B virus surface antigen	Negative
Hepatitis C virus antibody	Negative
Hantavirus antibody	Negative
Respiratory (5)	
Nasopharyngeal swab specimen for respiratory virus panel¶	Negative
BAL of RML for respiratory virus panel¶	Negative
BAL of LUL for respiratory virus panel¶	Negative
Brain tissue (10)	
MVEV RT-PCR and tissue culture#	
Corpus callosum	Positive
Upper spinal cord	Positive
Thalamus	Positive

*BAL, bronchoalveolar lavage; LUL, left upper lobe; MVEV, Murray Valley encephalitis virus; ND, not determined: RML, right middle lobe; RT-PCR, reverse transcription PCR. †Pan-flavivirus RT-PCR and sequencing of amplicon (*3*). ‡Arbovirus antigen standard panel: St. Louis encephalitis, Powassan, dengue, and West Nile viruses. §MVEV IgM ELISA positive, MVEV IgG ELISA negative, and MVEV plaque-reduction neutralization test negative (Centers for Disease Control and Prevention, Fort Collins, CO, USA). ¶TAG respiratory virus panel (Luminex Molecular Diagnostics, Inc., Toronto, Ontario, Canada), which tests for respiratory syncytial virus; human coronaviruses HKU1, OC43, NL63, and 229E; parainfluenza viruses 1, 2, 3, and 4; enteroviruses/rhinoviruses; human metapneumovirus; adenovirus; and influenza viruses A and B. #Pan-flavivirus RT-PCR and sequencing of amplicon (*3*). MVEV was isolated on Vero cells from fresh homogenates of biopsy specimens prepared at the time of autopsy on day of clinical illness.

During the ensuing 2 days, decerebrate posturing worsened, rigidity increased, and the patient became deeply comatose. Continuous electroencephalography monitoring showed onset of progressively worsening seizure activity refractory to phenytoin and levetiracetam. Infusions of intravenous diprivan and midazolam were required to produce burst-suppression (Technical Appendix Figure 1).

On day 8, a dilated, nonreactive right pupil developed. A CT scan of the brain showed marked thalamic hypodensity with sulcal effacement, acute obstructive hydrocephalus, and cerebellar tonsillar herniation (Figure 1, panel C). An emergent external ventricular drain was placed, and standard measures were taken to treat intracranial hypertension. Despite intervention, refractory intracranial hypertension developed (intracerebral pressure >70 mm H_2_O). A decompressive craniectomy was considered but the patient died on day 10 of worsening obstructive hydrocephalus (Figure 1, panel D) and cerebellar tonsillar herniation.

**Figure 2:**
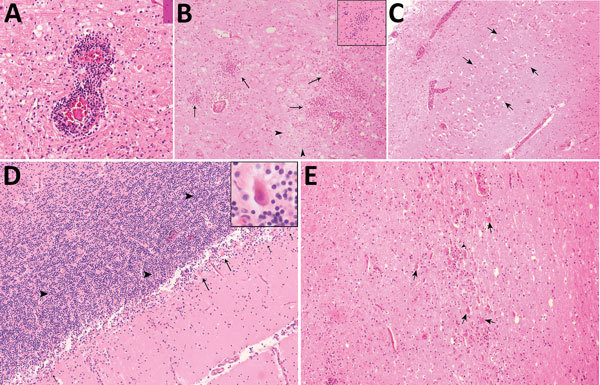
Hematoxylin and eosin–stained autopsy specimens from a patient with a fatal infection of Murray Valley encephalitis virus imported from Australia to Canada, 2011. A) Pons showing perivascular inflammatory infiltrate (original magnification ×40). B) Thalamus showing extensive inflammation (arrows) surrounding an area of rarefaction caused by necrosis (arrowheads) and neuronal loss (original magnification ×10); inset shows a microglial nodule (original magnification ×20). C) Pyramidal cell layer of the hippocampus showing extensive acute neuronal death (arrows) (original magnification ×4). D) Cerebellum showing severe depletion of Purkinje neurons and acute neuronal death (arrows and inset [original magnification ×40]) with relative sparing of the internal granule cell layer (arrowheads) and inflammation (short arrows) (original magnification ×10). E) Substantia nigra showing extensive inflammation, acute neuronal death (arrows), neuronophagia (arrowhead), and gliosis (original magnification ×10).

Autopsy showed severe active encephalomyelitis (Figure 2). Postmortem brain biopsy specimens from the corpus callosum, upper spinal cord, and thalamus were positive for MVEV by reverse transcription PCR (Technical Appendix Figure 2) with amplicon sequences identical to those obtained from CSF. MVEV was readily isolated on Vero cells from fresh homogenates prepared from each of the 3 biopsy specimens. The genomic sequence has been deposited in GenBank under accession no. KX229766. Additional autopsy findings included lymphocytic myocarditis, pulmonary edema, and acute tubular necrosis of the kidney. The liver and spleen were congested. The pancreas and ovaries were histologically normal.

## Conclusions

Historically, epidemics of MVE were recognized on the eastern coast of Australia; 6 known outbreaks were documented in the early twentieth century. Since the late 1970s, MVEV has largely been maintained in enzootic cycles involving mosquitoes and water fowl in the northern regions of Central and Western Australia; there have been multiple reported epidemics (*4*). Before the outbreak in 2011, heavy rains across Australia created ideal conditions for *Culex annulirostris* mosquitoes, the vector of MVEV, thus intensifying transmission to humans throughout the country (*5*). A shift in the demographic pattern of MVE cases toward non-Aboriginal, adult workers and tourists engaged in high-risk activities for mosquito exposure was observed (*1*). Australian States and Territories routinely use MVEV surveillance methods (mosquito monitoring, virus isolation from mosquitoes, sentinel chicken flocks, and climate surveillance) (*6*). Each state and territory has its own public health response and communications strategy (*1*), which target tourists to various degrees.

Human infection with MVEV is generally asymptomatic or mild with nonspecific symptoms, including headache, myalgia and, less commonly, rash. MVE is estimated to occur in <0.1% of infected persons but has a mortality rate of 15%–30% and produces long-term neurologic sequelae in <50% of survivors (*7*–*9*). Several distinct clinical patterns of MVE have been observed: encephalitis with complete recovery; cranial nerve/brainstem involvement with tremor; spinal cord involvement (poliomyelitis-like); and relentless progression to death, as seen in the patient we report (*8*). The presence of widespread magnetic resonance imaging abnormalities of the thalamus, midbrain, spinal cord, and cerebellum during acute illness predicted a devastating neurologic outcome (*10*). A novel feature of this case was the postmortem finding of viral myocarditis, which could account for early and unexpected respiratory decompensation of the patient.

Despite increased awareness of MVE, imported cases in Europe, Asia, and the Americas are rare (*11*). This case serves as a cautionary reminder of other viral etiologies of encephalitis that should be considered for returning travelers, although many of these etiologies might be outside the diagnostic capability of many clinical laboratories. Appropriate samples should be referred to centers in which specialized testing is available.

Technical Appendix Additional information on a fatal infection with Murray Valley encephalitis virus imported from Australia to Canada, 2011.
