# Supplementary material for: Fatal Infection with Murray Valley Encephalitis Virus Imported from Australia to Canada, 2011
Source: Emerg Infect Dis. 2017 Feb;23(2):280–3. doi: 10.3201/eid2302.161161 (PMC5324805; doi:10.3201/eid2302.161161)
Supplement: Technical Appendix — Additional information on a fatal infection with Murray Valley encephalitis virus imported from Australia to Canada, 2011. [file 16-1161-Techapp-s1.pdf]

Article DOI: http://dx.doi.org/10.3201/eid2302.161161

Fatal Infection with Murray Valley Encephalitis Virus Imported from Australia to Canada, 2011

Technical Appendix

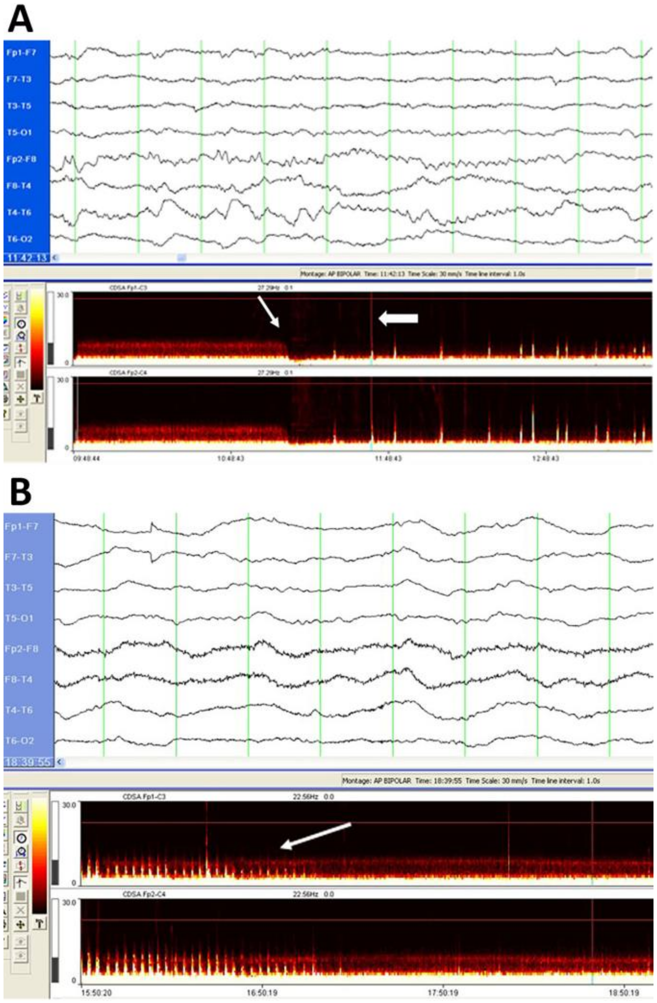

Technical Appendix Figure 1. Results of continuous electroencephalography (EEG) for a patient with a fatal infection of Murray Valley encephalitis virus imported from Australia to Canada, 2011. A) Raw EEG (top) and color density spectral array (CDSA) map (bottom) are provided. Small arrow indicates discontinuation of intravenous diprivan for neurologic assessment. Large arrow indicates onset of right temporal electrographic seizures, as shown in the raw EEG tracing. B) Arrow indicates time of reinitiation of diprivan and loading with intravenous phenytoin.

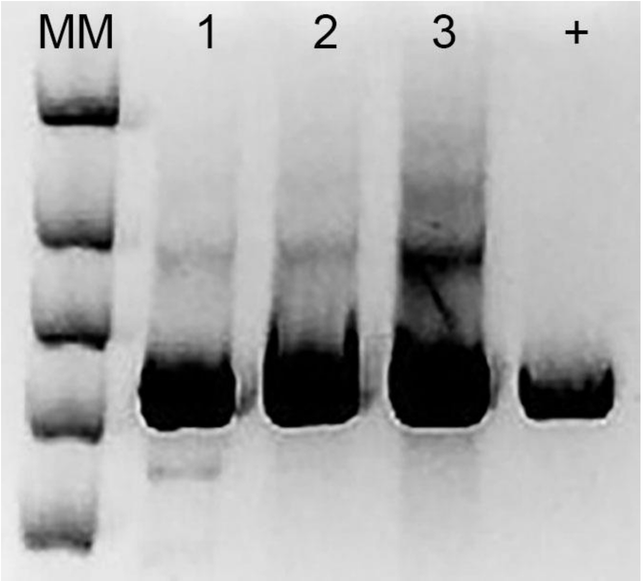

Technical Appendix Figure 2. Pan-flavivirus reverse transcription PCR performed for nucleic acids extracted from central nervous system tissue biopsy specimens obtained at autopsy from a patient with a fatal infection of Murray Valley encephalitis virus imported from Australia to Canada, 2011. Amplicons were sequenced (see text). Lane 1, MM, molecular mass ladder (FlashGel DNA Marker, 50 bp to 1.5 kb; Lonza, Basel, Switzerland); lane 1, corpus callosum; lane 2, upper spinal cord; lane 3, thalamus; lane +, flavivirus-positive control (expected amplicon size 854–863 bp depending on virus species).
